# Draft genomes of three abundant bacterial isolates from hypersaline Don Juan Pond, Antarctica

**DOI:** 10.1128/mra.00279-25

**Published:** 2025-12-29

**Authors:** Jacob M. C. Shaffer, Abigail Jarratt, Bruce W. Boles, Jill A. Mikucki

**Affiliations:** 1 Department of Microbiology, University of Tennessee – Knoxville 189504, Knoxville, Tennessee, USA; University of Southern California, Los Angeles, California, USA

**Keywords:** Antarctic extremophiles, heterotrophic bacteria, polar desert, CaCl_2_ brines

## Abstract

Calcium chloride-rich brines are extremely rare; thus, microbes inhabiting these systems can provide insight into bacterial adaptation to hypersalinity, low water activity, and chaotropicity. We present the genomes of three bacterial isolates from the sediments of Don Juan Pond, Antarctica, including members of the phyla Bacillota and Pseudomonadota.

## ANNOUNCEMENT

Don Juan Pond (DJP) is a hypersaline, CaCl_2_-rich brine within Wright Valley, Antarctica (77.565 S, 161.174 E; 1). DJP is one of the most saline brines on the planet and is of interest for studies seeking to describe the limits of life on our planet and as an analog feature for possible habitable niches on other worlds (2–4). Brine-saturated sediments below the pond water column were aseptically collected in November 2018 using sterile scoops and a push-corer (5). Representatives of three relatively abundant bacterial genera from this system were isolated from these materials.

Viable colonies were isolated from sediment (~1 g) with the addition of Difco Marine Broth 2216 (10:1 slurry), followed by gently shaking (100 rpm) to dislodge cells, centrifugation (50 × g, 1 h), and plating supernatant on Difco Marine Agar 2216. Plates were incubated at 10°C for 7 days; colonies with unique phenotypes were selected and serially passaged to ensure isolation. Cells were examined microscopically to assess culture purity and identified taxonomically via Sanger sequencing of the 16S rRNA gene as per Shaffer et al. (6; Table 1). Three isolates were selected for genomic sequencing based on relative abundance within a sediment 16S rRNA amplicon library (5). Strain DJP18 originated from a surficial sediment sample, and strains DJP31 and DJP39 were isolated from subsections of a sediment core, originating 16–18 and 14–16 cm from the surface, respectively. DJP18 was taxonomically placed in the genus *Loktanella* (Rhodobacteraceae), DJP31 in *Bacillus* (Bacillaceae), and DJP39 in *Virgibacillus* (Amphibacillaceae).

**TABLE 1:** Summary statistics of DJP draft genomes^*a*^

Genome	Isolation site	Closest relative by 16S rRNA gene (NCBI accession; % similarity)^*b*^	N paired reads	QUAST				CheckM			CoverM	GTDB-tk		PGAP
				Genome size	# Contigs	N50	GC content (%)	Comp.	Contam.	Strain hetero-geneity	Average coverage	Assigned classification	ANI to closest placement	CDS
*Loktanella* sp. DJP18	Surficial sediments	*Loktanella salsilacus* strain R-8904 (NR_ 025539.1; 98.31%)	20,857,526	5,067,553	423	5,709	62.31	99.09	2.6	0	99.57	d__Bacteria; p__Pseudomonadota; c__Alphaproteobacteria; o__Rhodobacterales; f__Rhodobacteraceae; g__*Loktanella*; s__	NA^*c*^	5,002
*Bacillus* sp. DJP31	Push core, 16–18 cm depth	*Bacillus mesophilus* strain SA4 (NR_ 149175.1; 96.54%)	3,686,914	4,275,924	117	142,071	37.17	97.7	2.25	0	99.32	d__Bacteria; p__Bacillota; c__Bacilli; o__Bacillales; f__SA4; g__*Bacillus*_BS; s__	NA^*c*^	4,256
*Virgibacillus* sp. DJP39	Push core, 14–16 cm depth	*Virgibacillus necropolis* strain LMG 19488 (NR_ 025472.1; 97.17%)	20,339,363	4,217,496	212	47,417	36.99	100	1.33	0	99.47	d__Bacteria; p__Bacillota; c__Bacilli; o__Bacillales_D; f__Amphibacillaceae; g__*Virgibacillus*_F; s__	79.27 (*Virgibacillus necropolis,* GCF_ 002224365.1)	4,199

^*a*^ Comp. - completeness; Contam. - contamination; CDS - coding sequences.

^*b*^ Performed using PCR-amplified 16S rRNA gene sequences.

^*c*^ NA, not assigned to terminal branch by GTDB-tk.

Cells were grown in Marine Broth at 10°C to an optical density (600 nm) of 0.8 and pelleted. DNA was extracted from cell pellets using a cetrimonium bromide buffer as per Campen (7; average DNA concentration 47 ng μL^−1^, determined by fluorometer). Library preparation was performed using a Nextera DNA Flex Library Preparation Kit (Illumina), and library quality was determined via an Agilent 2100 Bioanalyzer. Libraries were diluted to 0.6 nM before sequencing via Illumina NovaSeq (2 × 250; 500 cycles). Sequencing adapters were trimmed and low-quality reads removed using Trimmomatic v0.36 (8), and reads were assembled via Unicycler v0.4.8 (9). Assembly quality and coverage were assessed using QUAST v5.2.0 (10), CheckM v1.1.3 (11), and CoverM v0.7.0 (12). Resulting assemblies were taxonomically placed via GTDB v2.3.2 (13). Genomes were annotated via PGAP v6.10 (14). Default parameters were used for all software.

Genome results are summarized in Table 1. Isolate identities were based on the similarity of PCR-amplified 16S rRNA genes to the closest related type strains; isolates were not identified to species level via average nucleotide identity. All genomes are in draft form, with between 215 and 733 contigs per assembly. Genome completeness was estimated to be between 97.7–100%. DJP31 and DJP39 had a low GC content, at ~37%, while DJP18 had a GC content of ~62%. DJP39 had the smallest genome size, with 4.24 Mb and 4,199 predicted coding sequences, and DJP18 had the largest, with 5.14 Mb and 5,002 predicted coding sequences.

## Data Availability

Sequences for this project have been deposited in GenBank as a part of BioProject PRJNA1235758. Assembled genomes are deposited under the following accession numbers: *Loktanella* sp. DJP18: JBMERN000000000; *Bacillus* sp. DJP31: JBMERO000000000; *Virgibacillus* sp. DJP39: JBMERP000000000. Raw sequences have been deposited under accession numbers SRX28417067, SRX28417068, and SRX28417069 for DJP18, DJP31, and DJP39, respectively.
